# Comprehensive integrated analysis of MR and DCE-MR radiomics models for prognostic prediction in nasopharyngeal carcinoma

**DOI:** 10.1186/s42492-023-00149-0

**Published:** 2023-12-01

**Authors:** Hailin Li, Weiyuan Huang, Siwen Wang, Priya S. Balasubramanian, Gang Wu, Mengjie Fang, Xuebin Xie, Jie Zhang, Di Dong, Jie Tian, Feng Chen

**Affiliations:** 1 Beijing Advanced Innovation Center for Big Data-Based Precision Medicine, School of Medicine and Engineering, Beihang University, Beijing, 100191 China; 2 CAS Key Laboratory of Molecular Imaging, Institute of Automation, Chinese Academy of Sciences, Beijing, 100190 China; 3 Department of Radiology, Hainan General Hospital (Hainan Affiliated Hospital of Hainan Medical University), Haikou, Hainan 570311 China; 4 School of Artificial Intelligence, University of Chinese Academy of Sciences, Beijing, 100049 China; 5 Department of Psychiatry, Weill Cornell Medicine, New York, NY 10065 USA; 6 Department of Radiotherapy, Hainan General Hospital (Hainan Affiliated Hospital of Hainan Medical University), Haikou, Hainan 570311 China; 7 Department of Radiology, Kiang Wu Hospital, Santo António, Macao 999078 China; 8 Department of Radiology, Zhuhai People’s Hospital (Zhuhai Hospital Affiliated With Jinan University), Zhuhai, Guangdong 519000 China; 9 Engineering Research Center of Molecular and Neuro Imaging of Ministry of Education, School of Life Science and Technology, Xidian University, Xi’an, Shaanxi 710126 China; 10 Zhuhai Precision Medical Center, Zhuhai People’s Hospital, Zhuhai, Guangdong 519000 China

**Keywords:** Dynamic contrast-enhanced magnetic resonance imaging, Magnetic resonance imaging, Radiomics, Prognostic prediction

## Abstract

Although prognostic prediction of nasopharyngeal carcinoma (NPC) remains a pivotal research area, the role of dynamic contrast-enhanced magnetic resonance (DCE-MR) has been less explored. This study aimed to investigate the role of DCR-MR in predicting progression-free survival (PFS) in patients with NPC using magnetic resonance (MR)- and DCE-MR-based radiomic models. A total of 434 patients with two MR scanning sequences were included. The MR- and DCE-MR-based radiomics models were developed based on 289 patients with only MR scanning sequences and 145 patients with four additional pharmacokinetic parameters (volume fraction of extravascular extracellular space (*v*_*e*_), volume fraction of plasma space (*v*_*p*_), volume transfer constant (*K*^*trans*^), and reverse reflux rate constant (*k*_*ep*_) of DCE-MR. A combined model integrating MR and DCE-MR was constructed. Utilizing methods such as correlation analysis, least absolute shrinkage and selection operator regression, and multivariate Cox proportional hazards regression, we built the radiomics models. Finally, we calculated the net reclassification index and C-index to evaluate and compare the prognostic performance of the radiomics models. Kaplan-Meier survival curve analysis was performed to investigate the model’s ability to stratify risk in patients with NPC. The integration of MR and DCE-MR radiomic features significantly enhanced prognostic prediction performance compared to MR- and DCE-MR-based models, evidenced by a test set C-index of 0.808 vs 0.729 and 0.731, respectively. The combined radiomics model improved net reclassification by 22.9%–52.6% and could significantly stratify the risk levels of patients with NPC (*p* = 0.036). Furthermore, the MR-based radiomic feature maps achieved similar results to the DCE-MR pharmacokinetic parameters in terms of reflecting the underlying angiogenesis information in NPC. Compared to conventional MR-based radiomics models, the combined radiomics model integrating MR and DCE-MR showed promising results in delivering more accurate prognostic predictions and provided more clinical benefits in quantifying and monitoring phenotypic changes associated with NPC prognosis.

**Supplementary Information:**

The online version contains supplementary material available at 10.1186/s42492-023-00149-0.

## Introduction

As an endemic common malignancy, nasopharyngeal carcinoma (NPC) ranks first among head and neck malignant tumors with an incidence rate of 3.0 per 100000 individuals in Southeast Asia [1]. Radiotherapy-based comprehensive treatment is regarded as the standard strategy for NPC. Although the prognosis has considerably improved with the popularization of intensity-modulated radiotherapy in the past decades, the 5-year survival rate remains approximately 60% in patients with locoregional advanced NPC [2]. The lack of responses to therapy, recurrence, and distant metastasis are the main causes of poor prognosis in NPC [3]. Pretreatment identification of adverse events is thus important to make individualized treatment decisions. In current clinical practice, tumor therapeutic response is only evaluated using RECIST (one-dimensional descriptors) or WHO (two-dimensional descriptors) criteria [4]. These criteria have significant potential in indicating therapeutic response but often fail to predict progression-free survival (PFS). The most commonly used benchmark for prognostic estimation of NPC is the tumor-node-metastasis (TNM) staging system. However, patients with similar treatment regimens or at the same stage can show large variations in clinical outcomes [5]. Thus, the present TNM staging system may not provide adequate prognostic information to comprehensively express the biological heterogeneity of NPC [6].

Magnetic resonance (MR) imaging plays an important role in NPC detection and staging. The excellent soft-contrast resolution of conventional MR can reveal the morphological features. Dynamic contrast-enhanced MR (DCE-MR) is an MR perfusion technique that consists of a series of rapid contrast-enhanced T1-weighted (CET1-w) acquisitions of serial MR images with high temporal resolution before and after the administration of clinically available contrast agents (i.e., gadolinium chelates). These contrast agents alter the signal intensity in the target tissues, which is proportional to their concentration. During analysis, signal intensities are converted into concentration curves, provided that a pre-contrast relaxation map is obtained and the relationship between signal intensity and concentration is known for the specific MR sequence used. With appropriate modeling, quantitative analysis allows for the inference of blood flow, blood volume, and vascular permeability, in addition to the morphological tumor characteristics used in clinical practice. The pharmacokinetic parameters of DCE-MR, such as volume fraction of extravascular extracellular space (*v*_*e*_), volume fraction of plasma space (*v*_*p*_), volume transfer constant (*K*^*trans*^), and reverse reflux rate constant (*k*_*ep*_), can potentially reflect angiogenesis and tumor aggressiveness [7]. Until now, many studies have utilized DCE-MR to predict therapeutic response or prognosis in patients with NPC [8–10]. However, the concentration of the contrast agent in the vessel and the variability of arterial input function (AIF) have been considered the main reasons for the low dependability of DCE-MR [11–13].

Radiomics is a popular medical image analysis technique that involves image processing, feature engineering, and deep learning algorithms, which can extract features related to lesion shape, statistics, and texture and construct a mathematical model associated with clinical events [14–17]. In recent years, radiomics has significantly assisted clinicians in the early diagnosis, determination of treatment plans, and prognostic assessment of NPC [18–22]. Due to its fast speed, accurate calculation, and noninvasiveness, radiomics presents immense prospects for development. However, some unresolved issues, such as interpretability, are becoming serious obstacles. In most deep learning models, class activation mapping (CAM)-based methods [23, 24] can be used to generate visual explanation maps. However, for feature engineering-based radiomics models, the algorithm for generating radiomics feature maps still needs to be improved to present noteworthy regions inside the tumor. In previous radiomics studies, most of the predefined radiomic features tended to be calculated based on the global region of interest (ROI), while the visual representation of these features requires individual calculations from each local image patch and then overlaying the feature values ​​of all patches onto the ROI [25]. Therefore, the image patch size may significantly affect the visual representation of radiomic features. The statistical calculation of radiomic features tends to be more accurate when a larger local image patch is selected; however, the resolution of the visualized image may be reduced accordingly. There is a compromise between accurate feature extraction and precise information localization, which restricts the patch size and step size settings.

Radiomics can help evaluate tumor heterogeneity and the microenvironment, and thus lead to the identification of novel predictors of prognosis [26, 27]. Radiomic analysis based on multiparametric MR has been successfully performed to predict individual PFS in patients with advanced NPC [28, 29]. Moreover, the radiomic features derived from MR images are useful for predicting the treatment response to chemoradiotherapy [30] and induction chemotherapy [31, 32] in patients with NPC. However, most previous studies have focused on conventional MR sequences. Recent studies have revealed that DCE-MR-based radiomics are more efficient than conventional MR sequences in predicting prognosis and evaluating treatment responses in malignant gliomas [33], breast cancer [34], and rectal cancer [35]. In addition, the prognostic predictive performance of MR-based radiomics models has been reported to outperform traditional clinical models in previous radiomics studies [36–38]. In this study, we developed a radiomics model by combining MR and DCE-MR imaging features to predict PFS and assessed its incremental value in MR- and DCE-MR-based radiomics models. To test our hypothesis that MR-based radiomics features contain prognostic information related to the underlying angiogenesis and pharmacokinetic information, we further visualized MR-based radiomics feature maps on DCE-MR images.

## Methods

**Fig. 1:**
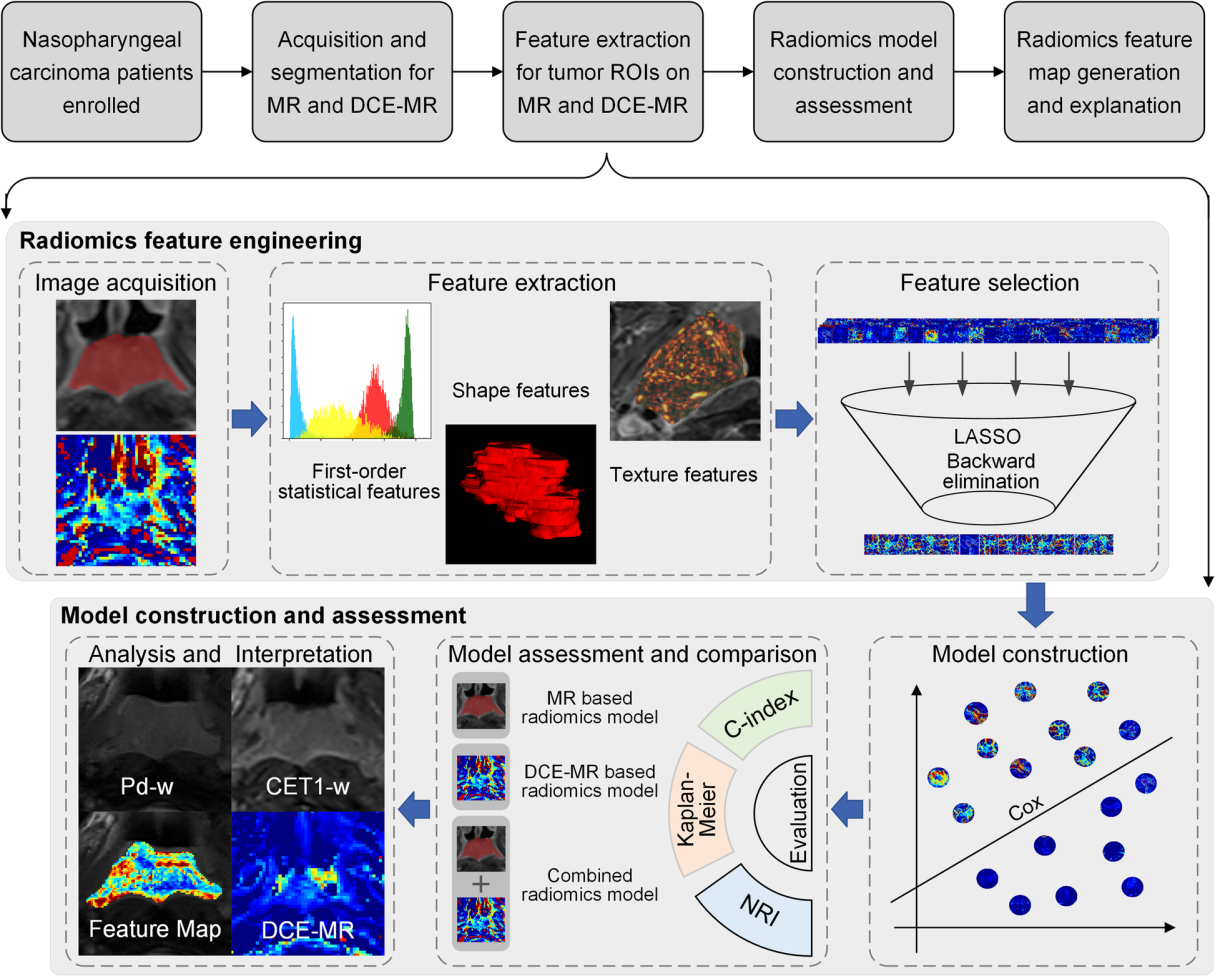
Study layout. The MR based and DCE-MR based prognostic prediction models were constructed after image acquisition, feature extraction, and feature selection. The models were then assessed by C-index, Kaplan-Meier survival curve analysis, and NRI. Finally, four DCE-MR parameters and the radiomics feature map of MR based model were shown and analyzed

This study was approved by the Institutional Review Board of Hainan General Hospital, which waived the requirement for written informed consent. The overall workflow includes patient enrollment, segmentation for MR and DCE-MR images, radiomics feature extraction, radiomics model construction and assessment, as shown by the flowchart in Fig. 1. The open source PyRadiomics platform (version 3.0.1) was used in this study, which adheres to the image biomarker standardization initiative protocol.

### Study population

**Fig. 2:**
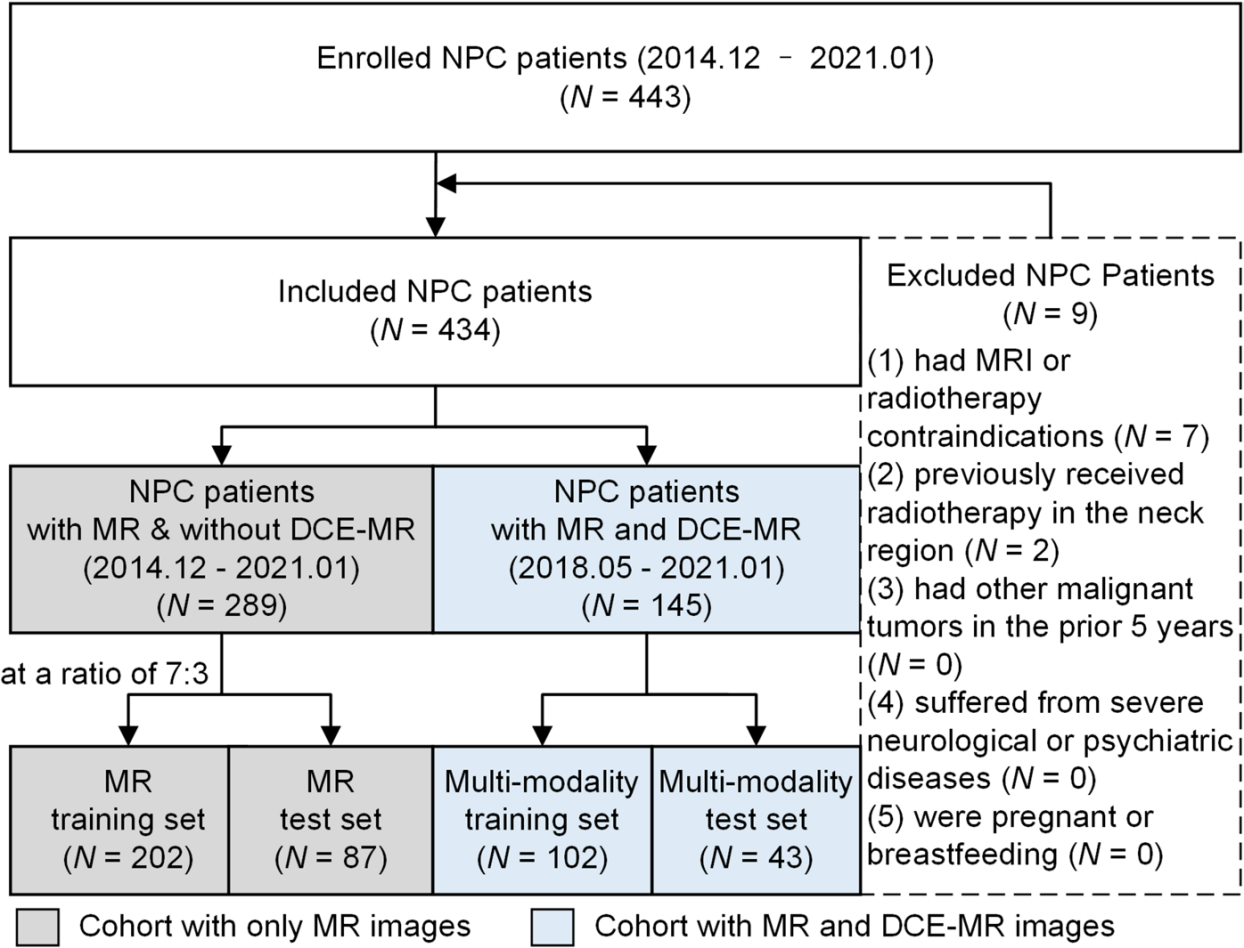
Patient recruitment flowchart. A total of 434 patients were included in this study. The patients were then divided into two cohorts based on the presence or absence of DCE-MR images. The MR cohort with only MR images was randomly divided into an MR training set and test set at a ratio of 7:3. In addition, the multi-modality cohort with both MR and DCE-MR images was randomly allocated to a multi-modality training set and test set at a ratio of 7:3 as well. The MR cohort shown in the gray box and the multi-modality cohort in blue box constituted the whole dataset of this study

This study enrolled patients with NPC from December 2014 to January 2021, and the inclusion and exclusion criteria can be found in Supplementary Methods 1. As shown in Fig. 2, we first divided the patients into two cohorts based on whether they had corresponding DCE-MR images: the cohort with only MR images (*n* = 289) was allocated to an MR training set and an MR test set at a ratio of 7:3. Another cohort with both MR and DCE-MR images (*n* = 145) was allocated to a multimodality training set and a multimodality test set. An MR-based model was built on the MR training set and evaluated on the MR test set, and a DCE-MR-based model was built on the multimodality training set and evaluated on the multimodality test set. Because the multi-modality training and test sets contained not only DCE-MR images but also MR images, a combined model integrating the MR-based model and DCE-MR features was constructed and evaluated on these two sets.

### Acquisition and segmentation for MR and DCE-MR

MR examinations were conducted using a 3.0 T MR system (Siemens Skyra, Erlangen, Germany) with a 20-channel head/neck coil. The detailed protocol can be found in Supplementary Methods 2. The pharmacokinetic parameters (*v*_*e*_, *v*_*p*_, *K*^*trans*^, and *k*_*ep*_) of DCE-MR were estimated using the extended Tofts’ linear model, with the intracranial internal carotid artery serving as the AIF. The three-dimensional ROIs of the primary lesions were manually delineated on proton density-weighted (PD-w) images, CET1-w images, and DCE-MR images slice-by-slice using ITK-SNAP (version 3.6.0, http://www.itksnap.org). The segmented ROIs were created by a junior radiologist (G.D. with 3-year experience) and further checked by a senior radiologist (W.H. with 12-year experience), both of whom had no access to patients' clinical information.

### Clinical endpoint and follow-up

Patients were assessed by MR examination every 3 months for the first 24 months, and then every 6 months. The endpoint of our study was PFS, which was defined as the time from the initial treatment to the first recurrence of the disease, death caused by NPC, or the latest follow-up visit, whichever occurred first. Disease progression was identified using pathological biopsy and/or imaging methods.

### Image pre-processing and radiomics feature extraction

Based on the open-source PyRadiomics platform (version 3.0.1), we extracted three types of radiomics features for MR and DCE-MR images, including shape, first-order statistics, and texture features [39]. The shape features were only calculated on the original images (i.e., tumor ROIs). The texture features and first-order statistical features were also extracted from the derived images using filters. Because the MR signal is relative, with substantial differences between scanners and vendors, we normalized the image before the matrix-based texture feature calculation to reduce the confounding effect. Here, we performed gray-value discretization using the window width of the five MR intensity values. The details of radiomics feature engineering can be found in Supplementary Methods 3.

### Construction and evaluation of MR based radiomics models

To identify a subset of features associated with PFS, we employed the least absolute shrinkage and selection operator (LASSO) method for radiomics feature selection on the MR training set using the ‘glmnet' package in the R language. The selection of the λ parameter was performed based on tenfold cross-validation and the minimum error criterion. We then conducted backward feature elimination based on the *p* values in multivariate regression analysis. Finally, a multivariate Cox proportional hazards (CPH) regression model was used to build the radiomics model by fitting the aforementioned radiomic features. To address the issue of multicollinearity, we employed a variance inflation factor (VIF) function (‘car' package in R language) to assess the presence of multicollinearity among all the feature considered in this study. Specifically, a VIF exceeding 5 was considered an indicator of multicollinearity. In this study, three MR-based radiomics models were constructed for PD-w images, CET1-w images, and their combinations (multi-sequence MR images). The model with the best prognostic prediction performance was selected for further comparison.

C-index was calculated to assess the prognostic performance of each model. In addition, Kaplan-Meier survival analysis was performed to investigate the ability of each model in risk stratification of patients with NPC, and a log-rank test was conducted to determine the statistical differences between the two risk groups.

### Construction and evaluation of DCE-MR based radiomics models

In the multi-modality training set, five DCE-MR-based radiomics models were constructed using images of four DCE-MR parameters (*v*_*e*_, *v*_*p*_, *K*^*tran*^, and *k*_*ep*_) and their combination (multi-parameter DCE-MR-based model), following the same procedure as the MR-based radiomics models. In the construction of the multiparameter DCE-MR-based model, given the substantial interrelation among the numerous features in our original dataset, we initially employed Pearson correlation analysis to eliminate redundant features. Following this, consistent with the construction of MR-based models, we sequentially implemented LASSO regression and backward feature elimination for additional feature dimensionality reduction. Finally, the CPH regression was used to construct a survival analysis model. In Pearson correlation analysis, we quantified the degree of similarity between pairwise features. Features that displayed a correlation coefficient exceeding 0.8 were deemed to have significant similarity. In these instances, one feature was arbitrarily excluded, thereby mitigating the covariance between features to a certain degree. DCE-MR-based radiomics models were evaluated using the same methods as the MR-based radiomics models in multimodality training and test sets.

### Combined model based on MR and DCE-MR

To explore whether the DCE-MR radiomic features could improve the performance of the MR-based radiomics model, a combined model was constructed in a multimodality training set by integrating the DCE-MR radiomic features and an optimal MR-based radiomics model. Pearson’s correlation analysis was first performed to eliminate redundant DCE-MR radiomic features. To ensure that the DCE-MR-based model captured tumor information divergent from the MR-based model, features highly correlated (Pearson correlation analysis) with the MR-based radiomics model were eliminated. This refinement ensures an enhanced performance upon the fusion of both models. Finally, we incorporated the remaining DCE-MR radiomics features and MR-based radiomics model predictions and adopted LASSO regression and backward feature elimination to determine the PFS-related feature subset. Similarly, a multivariate CPH regression model was used to construct a combined model.

We calculated the C-index and conducted Kaplan-Meier survival curve analysis to evaluate the prognostic performance and risk stratification ability of the combined model in the DCE-MR test set. Additionally, the net reclassification index (NRI) was calculated to quantify the relative improvement of the combined model in prognostic prediction compared with the MR- and DCE-MR-based radiomics models.

### MR based radiomics feature map

To extract features of the tumor ROIs more accurately, as well as to maintain a high resolution of the feature map, a sliding window algorithm with a larger window width (patch size) and a smaller step size was applied to calculate the radiomic features of image patches pixel by pixel. A larger window width (patch size) improves the accuracy of feature computation. However, a smaller step size (1 in this study) allows us to generate the final feature map with a higher image resolution. In this study, the length of the shortest edge of the outer rectangles of the tumor ROIs was 21. Consequently, the window size should be smaller than 21 to ensure that radiomic features are extracted from the inside area of the tumor. In addition, to obtain a relatively large window width such that the extracted information could be more accurate, the window width was set to 21. It is worth highlighting that there is a compromise between too small a window size, which leads to inaccurate feature calculation, and too large a window size, which leads to excessive aggregation of information. Owing to the limitations of the tumor ROI size in this study, the optimal window size in practice needs to be investigated in more detail in future studies. Feature extraction was performed at the image patch level, and the feature values were concatenated according to the patch coordinates to form a feature matrix. We then overlaid the feature matrix onto the ROI of the MR images of PD-w/CET1-w to generate an MR-based radiomics feature map. A more detailed generation process for the MR-based radiomics feature map is provided in Supplementary Methods 4.

## Results

### Patient characteristics and clinical outcomes

A total of 443 consecutive patients newly diagnosed with NPC were enrolled, and 434 patients were included after verifying the exclusion criteria. The mean age was 49.13 ± 11.56 years. The median PFS was 27 months (range, 3–75 months). During follow-up, 70 patients showed disease progression. The patient characteristics in the MR and multimodality cohorts are summarized in Table 1.

**Table 1:** Patient characteristics in MR and multi-modality cohorts

Characteristic	MR cohort		Multi-modality cohort	
	**Training set (*n* = 202)**	**Test set (*n* = 87)**	**Training set (*n* = 102)**	**Test set (*n* = 43)**
Sex				
Male	144 (71.3%)	65 (74.7%)	75 (73.5%)	29 (67.4%)
Female	58 (28.7%)	22 (25.3%)	27 (26.5%)	14 (32.6%)
Age				
Median (IQR)	48 (41–56)	49 (40–57)	49 (42–57)	51 (40–57)
≤ 40	50 (24.8%)	23 (26.4%)	19 (18.6%)	12 (27.9%)
40–50	67 (33.2%)	48 (55.2%)	63 (61.8%)	25 (58.1%)
> 50	85 (42%)	16 (18.4%)	20 (19.6%)	6 (14.0%)
T stage				
T1	7 (3.5%)	1 (1.2%)	3 (2.9%)	0 (0.0%)
T2	54 (26.7%)	32 (36.8%)	29 (28.4%)	6 (14.0%)
T3	84 (41.6%)	25 (28.7%)	48 (47.1%)	20 (46.5%)
T4	57 (28.2%)	29 (33.3%)	22 (21.6%)	17 (39.5%)
N stage				
N0	12 (5.9%)	6 (6.9%)	8 (7.8%)	1 (2.3%)
N1	49 (24.3%)	23 (26.4%)	28 (27.5%)	8 (18.6%)
N2	106 (52.5%)	47 (54.0%)	48 (47.1%)	19 (44.2%)
N3	35 (17.3%)	11 (12.7%)	18 (17.6%)	15 (34.9%)
M stage				
M0	199 (98.5%)	84 (96.6%)	100 (98.0%)	42 (97.7%)
M1	3 (1.5%)	3 (3.4%)	2 (2.0%)	1 (2.3%)
Overall stage				
I	2 (1.0%)	0 (0.0%)	0 (0.0%)	0 (0.0%)
II	18 (8.9%)	14 (16.1%)	11 (10.8%)	1 (2.3%)
III	93 (46.0%)	32 (36.8%)	57 (55.9%)	19 (44.2%)
IV	86 (42.6%)	37 (42.5%)	34 (33.3%)	23 (53.5%)
V	3 (1.5%)	4 (4.6%)	0 (0.0%)	0 (0.0%)
Follow-up time (mo)				
Median (IQR)	49 (23–61)	55 (34–63)	11 (7–17)	10 (6–19)

Independent-sample* t*-test was applied for continuous variables, and ${\chi }^{2}$ test was applied for categorical variables. No significant differences were found between the MR and multi-modality cohorts in terms of sex, age, T stage, and N stage (*p* = 0.315–0.987). The M stage, overall stage, and follow-up time differed significantly between the two cohorts (*p* = 2.96 × 10^–4^, 0.015, and 2.20 × 10^–16^, respectively). *IQR* interquartile range

### Construction and evaluation of MR based radiomics models

After data cleaning (deleting features with missing or repetitive values), 1681 and 1678 radiomic features were calculated based on the PD-w and CET1-w sequences, respectively. Two and four features were selected for the PD-w and CET1-w sequences, respectively, using LASSO-Cox regression. In addition, from the combination of features of the PD-w and CET1-w sequences, four radiomic features were screened out with significant multivariate regression coefficients (*p* < 0.05), including wavelet-LHL_glszm_LAHGLE and lbp_firstorder_Variance from the PD-w sequence, as well as wavelet-HHH_glszm_LAHGLE and squareroot_firstorder_RMAD from the CET1-w sequence. Finally, the PD-w-, CET1-w-, and multi-sequence MR-based radiomics models were constructed by fitting the selected features using multivariate CPH regression.

**Fig. 3:**
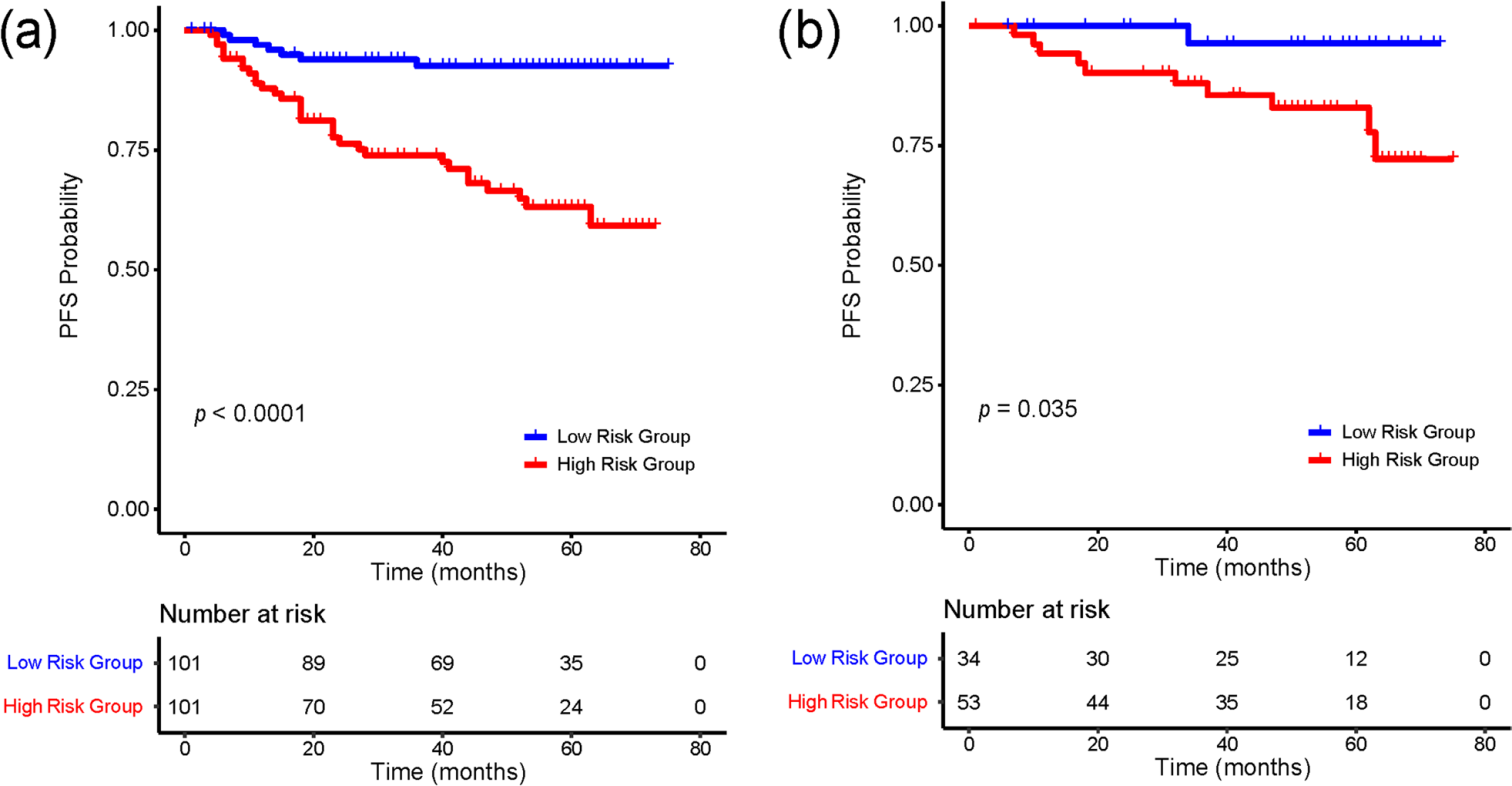
Kaplan-Meier survival curve analysis of multi-sequence MR based radiomics model on MR cohort. Kaplan-Meier survival curves of multi-sequence MR based radiomics model on (**a**) MR training set and (**b**) MR test set. A *p* value < 0.05 was used to determine whether the difference was statistically significant between the low- and high-risk groups

For the PD-w- and CET1-w-based radiomics models, the C-indices were 0.657 (95%CI: 0.549–0.765) and 0.664 (95% CI: 0.540–0.789) on the MR test set and 0.687 (95%CI: 0.582–0.791) and 0.626 (95%CI: 0.482–0.770) on the multi-modality test set, respectively. The multi-sequence MR-based radiomics model demonstrated the best prognostic prediction performance among the MR-based models, with a C-index of 0.729 (95%CI: 0.611–0.847) on the MR test set and 0.702 (95%CI: 0.547–0.857) on the multi-modality test set. On the MR training and test sets, all three MR-based models performed well in the risk stratification of NPC, with statistical significance (log-rank test, *p* < 0.05). The multi-sequence MR based radiomics model achieved superior risk stratification ability among the three MR-based models. The Kaplan-Meier survival curves of the multi-sequence MR-based radiomics model are shown in Fig. 3. We used the median output values of the radiomics model in the training set as a cutoff to stratify patients into high- and low-risk groups.

### Construction and evaluation of DCE-MR based radiomics models

We extracted 1392, 1395, 1391, and 1394 radiomic features from the images of the four pharmacokinetic parameters (*v*_*e*_, *v*_*p*_, *K*^*trans*^, and *k*_*ep*_). Similar to the feature selection and model construction processes of the PD-w- and CET1-w-based radiomics models, five, four, four, and six features were selected for *v*_*e*_, *v*_*p*_, *K*^*trans*^, and *k*_*ep*_ based models, respectively. Four DCE-MR parameter-based models were constructed. To construct the multiparameter DCE-MR-based radiomics model, three features extracted from *K*^*trans*^, *v*_*e*_, and *v*_*p*_ were determined using Pearson correlation analysis and LASSO regression.

Five DCE-MR-based models were evaluated in the multi-modality test set, with a C-index of 0.654 (95%CI: 0.493–0.815) for *K*^*trans*^ based model, 0.606 (95%CI: 0.378–0.834) for *v*_*e*_ based model, 0.587 (95%CI: 0.337–0.836) for *v*_*p*_ based model, 0.644 (95%CI: 0.458–0.830) for *k*_*ep*_ based model, and 0.731 (95%CI: 0.595–0.867) for the multi-parameter DCE-MR based model. Of the five models compared, the multi-parameter DCE-MR-based radiomics model achieved the highest C-index but still failed to significantly risk stratify NPC in the Kaplan-Meier survival curve analysis (*p* = 0.08).

### Construction and evaluation of combined radiomics model

**Fig. 4:**
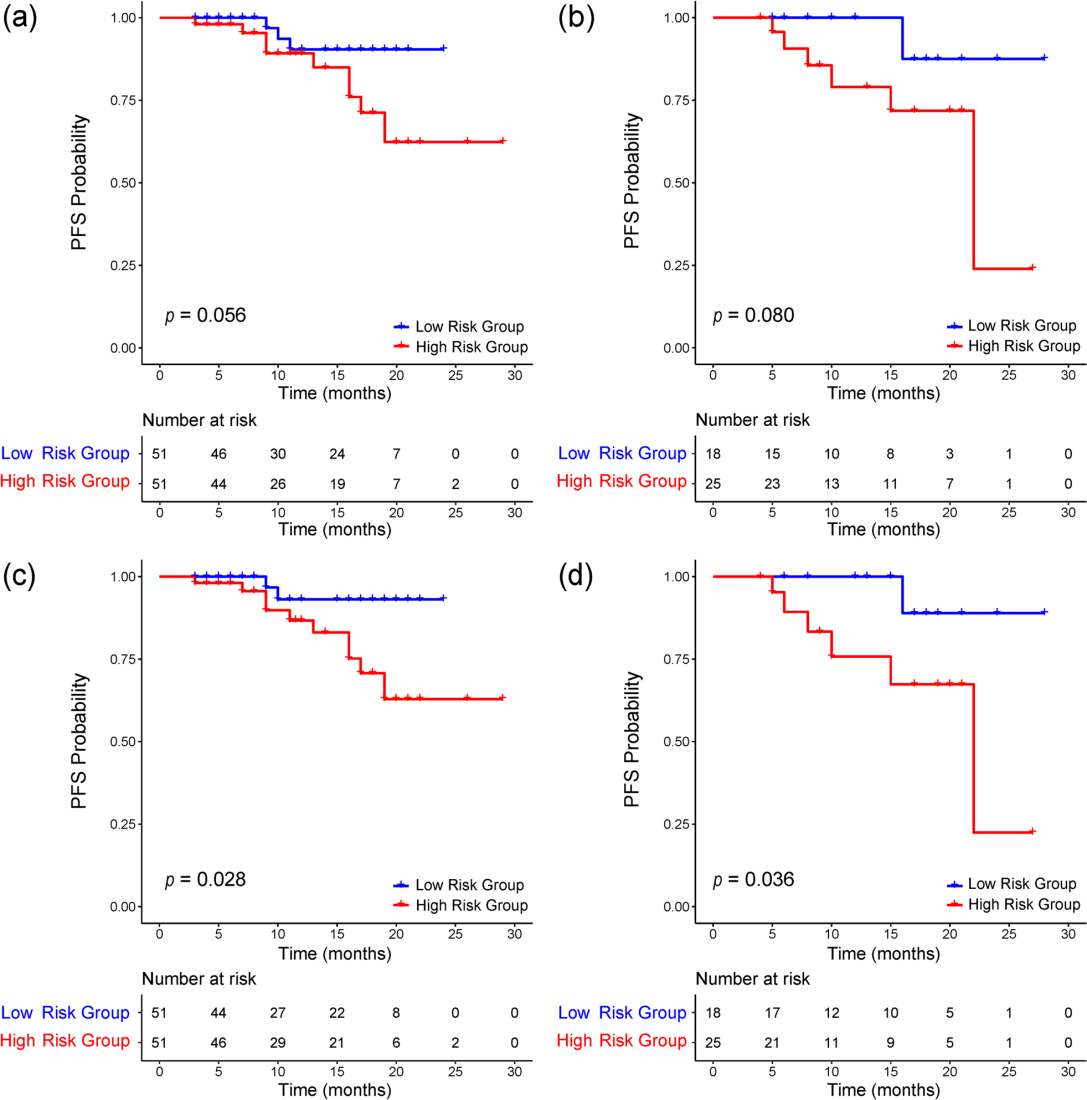
Kaplan-Meier survival curve analysis of DCE-MR based radiomics model and the combined radiomics model on multi-modality cohort. Kaplan-Meier survival curves of multi-parameter DCE-MR based radiomics model on (**a**) multi-modality training set and (**b**) multi-modality test set; and the combined radiomics model on (**c**) multi-modality training set and (**d**) multi-modality test set. A *p* value < 0.05 was used to determine whether the difference was statistically significant between the low- and high-risk groups

We constructed a combined model by integrating the radiomic features of DCE-MRI and a multi-sequence MR-based radiomics model. After Pearson correlation analysis and LASSO regression, five features were selected: two texture features extracted from *K*^*trans*^ (maximal correlation coefficient and gray level non-uniformity normalized, abbreviated as MCC and GLNN), two texture features extracted from *k*_*ep*_ (complexity and large dependence high gray level emphasis, abbreviated as complexity and LDHGLE), and the prediction of the MR-based radiomics model. The detailed feature selection flow and LASSO-CV regression process are shown in Supplementary Methods 3.5 and 3.6, respectively. By calculating the VIF, it was observed that all features had a VIF value below 5. The range of VIF values for the combined model and other models can be found in Supplementary Methods 3.7. This finding demonstrates that the constructed radiomics regression model is stable and is not significantly affected by high multicollinearity among the features. The C-index of the combined radiomics model achieved 0.812 (95%CI: 0.620–0.989) on the multi-modality training set, and 0.808 (95%CI: 0.691–0.924) on the multi-modality test set. As shown in Table 2, the combined radiomics model showed a statistically significant improvement in prognostic performance compared with all DCE-MRI- and MR-based models (*p* < 0.05). Kaplan-Meier survival analysis (Fig. 4c and d) demonstrated that the combined radiomics model could significantly stratify the risk of NPC in the multi-modality training set (*p* = 0.028) and multi-modality test set (*p* = 0.036). To verify the robustness of our combined model across different age groups, sexes, T stages, and N stages, we performed subgroup analyses of these clinical parameters. The outcomes highlight the consistent performance of the combined model across these subgroups, with C-index values ranging from 0.779 to 0.810; no significant differences were observed between the different subgroups. Finally, we quantified the improvement of the combined radiomics model in prognostic prediction by computing the NRI, where the NRI achieved 0.526 (combined model vs multi-sequence MR-based model, 95% CI: 0.265–0.706) and 0.229 (combined model vs multi-parameter DCE-MR-based model, 95%CI: 0.134–0.579).

**Table 2:** Performance of radiomics models on multi-modality test set

Model		C-index (95%CI)	*p*
MR based	PD-w	0.687 (0.582–0.791)	0.022
	CET1-w	0.626 (0.482–0.770)	0.032
	Multi-sequence MR	0.702 (0.547–0.857)	0.030
DCE-MR based	*K*^*trans*^	0.654 (0.493–0.815)	0.002
	*k*_*ep*_	0.644 (0.458–0.830)	0.013
	*v*_*e*_	0.606 (0.378–0.834)	0.030
	*v*_*p*_	0.587 (0.337–0.836)	0.027
	Multi-parameter DCE-MR	0.731 (0.595–0.867)	0.043
MR and DCE-MR	Combined	0.808 (0.691–0.924)	-

*CI* confidence interval, *p* < 0.05, significant differences in C-indices between the combined model and MR/DCE-MR-based models

### MR based radiomics feature map

**Fig. 5:**
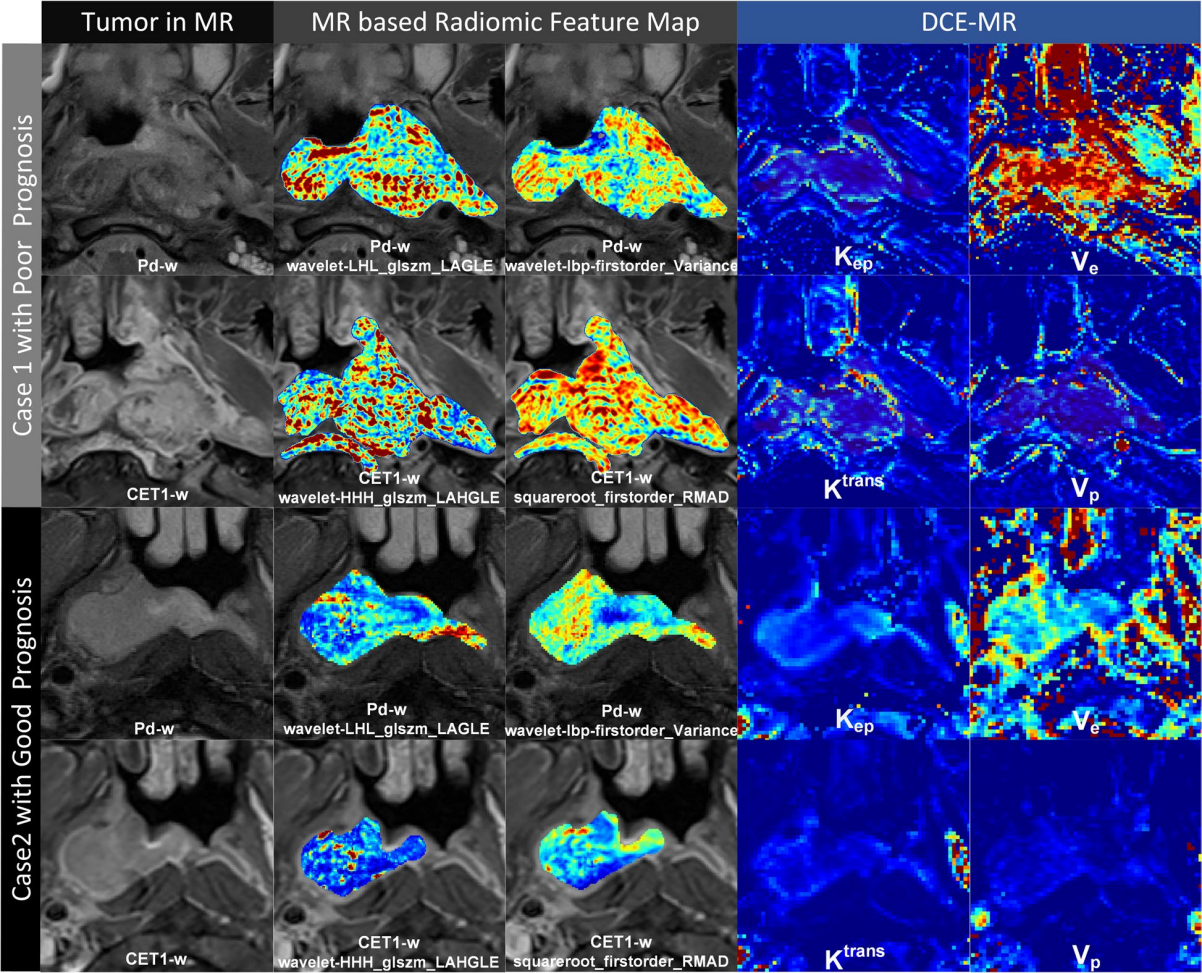
Typical examples of poor prognosis and good prognosis. Each case provides 10 subgraphs, including 2 MR images, 4 radiomics feature maps and 4 DCE-MR parameter images. The name of each subgraph is marked below itself

As shown in Fig. 5, a 41-year-old male patient with stage IV NPC (case 1) was confirmed to have metastasis at 3 months, and a 57-year-old male patient with stage IV NPC (case 2) did not experience any disease progression after treatment at 21 months. The tumor in case 1 showed a more obvious enhancement on CET1-w images and was more heterogeneous on PD-w images than that in case 2. DCE maps showed a similar tendency, with a poor prognosis case having higher perfusion and more heterogeneity in the tumor area. These characteristics were visualized to some extent using MR-based radiomics feature maps, particularly first-order feature maps (i.e., feature maps of lbp_firstorder_Variance in the PD-w sequence and squareroot_firstorder_RMAD in the CET1-w sequence). MR-based radiomics feature maps have the potential to be novel tools for visualizing tumor perfusion and heterogeneity. However, MR-based radiomics feature maps cannot reflect all the angiogenesis information of DCE-MR within tumors, which may also explain why DCE-MR features could help improve the performance of MR-based radiomics models in prognostic prediction.

## Discussion

The combined radiomics model established in this study outperformed the MR based radiomics models as well as DCE-MR based radiomics models in prognosis prediction. This is primarily because angiogenesis information within tumors in DCE-MR complements MR-based radiomic features. In conventional MR-based radiomics, the models generated from the combination of PD-w and CET1-w images demonstrated better prognostic performance than the model from either PD-w or CET1-w alone, and all MR-based radiomics models could significantly stratify the risk levels of patients with NPC. In addition, in DCE-MR-based radiomics models, the joint analysis of multiple parameters enhanced the efficiency compared to any single parameter model but was still unable to achieve significant risk stratification in patients with NPC. Furthermore, a high-resolution radiomics feature map was constructed to visualize and interpret the MR-based radiomic features, indicating that the MR-based radiomic features contained strong prognostic information, which might be interpreted using the underlying pharmacokinetic information quantitated by DCE-MR. Radiomic feature maps have the potential to visualize NPC prognosis to some extent.

Compared to traditional methods, radiomics provides a new train of thought for the potential associations between tumor angiogenesis and biological behaviors from a powerful and noninvasive perspective. Similar conclusions were reported in our previous study on advanced NPCs [29]. Only CET1-w and PD-w images were used to construct our radiomics models. Both the CET1-w and PD-w single-sequence models showed reliable capability in evaluating PFS, with the PD-w sequence model performing better. Their combination yielded significantly improved efficiency by integrating the morphological and functional features that affect the biological behavior of tumors. These results were similar to the naked-eye experience of radiologists in clinical practice. Unlike other tumors, the T staging of NPC mainly depends on the accurate tumor boundary information provided by the PD-w rather than the CET1-w sequence. However, CET1-w images can capture flow and heterogeneity information using a contrast agent, which helps solve the problem in which the signal of the tumor invading the nasopharynx muscle on PD-w images looks similar to that of inflammatory edema.

DCE-MR, as a non-invasive imaging modality, shows potential in reflecting blood volume, blood flow, and vascular permeability [40]. In Malamas et al.’s [41] study, significant reductions in tumor blood flow, vascularity permeability, and plasma volume fraction were observed on DCE-MR in colon tumors. Our previous study also indicated significant correlations of DCE-MR pharmacokinetic parameters with EGFR and Ki-67 expression levels in NPC patients [7]. Thus, DCE-MR can detect tumor angiogenesis and heterogeneity by quantifying pharmacokinetic parameters, so as to realize non-invasive treatment monitoring and predict prognosis [8, 10, 42]. Our results revealed that the MR based radiomics model demonstrated better performance in risk stratification in patients with NPC than the DCE-MR based radiomics model. This may be because fast dynamic enhancement sequences achieve multiphase repeated scanning within a short time. Notably, our combined model did not show an obvious advantage, as illustrated by the Kaplan-Meier survival curve analysis, which may be due to the small size of the test set. However, these findings require further validation.

An MR-based radiomics model was constructed using two first-order statistical and two textural features. The two texture features were both large-area high-gray-level emphasis (LAHGLE) but were estimated on the PD-w and CET1-w sequences, respectively. LAHGLE computes the proportion of the joint distribution of larger zones with higher grey-level values within the tumor, reflecting tumor heterogeneity. Both first-order statistical features (variance and robust mean absolute deviation) reflected the uniformity of gray values in the tumors, suggesting that a more homogeneous tumor on PD-w or CET1-w images may indicate a better prognosis. The MR-based radiomics model and four DCE-MR radiomic features (extracted from *K*^*trans*^ and *k*_*ep*_ images). For *K*^*trans*^, MCC measures the texture complexity and GLNN quantifies the similarity of pixel values. For *k*_*ep*_, complexity focuses on rapid changes in pixel values, and LDHGLE computes the distribution of large dependence with higher grey-level values [39].

In our study, we found that the MR-based radiomics feature map showed promising consistency with the DCE parameter maps in terms of the NPC tumor boundary and heterogeneity. Radiomic features related to NPC prognosis were also identified to be associated with tumor heterogeneity in MR images [43]. However, the interpretability of radiomics is slightly poor, in addition to its quantitative ability. Compared with artificially assessed radiological measurements, there is a vacancy in the visual interpretation of the selected radiomic features, which motivated our attempts to form a reasonable interpretative tool for clinical practice. This challenge has been well recognized, and some researchers have made efforts to find correlations between radiomic features and known biological markers, such as HIF [44] or fibroblast growth factor receptor [45]. However, the biological data are often difficult to acquire because of complex protocols, while radiomic features containing more spatial and functional information are conveniently available. Akram et al.’s study [46] revealed that there was a substantial difference in MR-based radiomic features extracted from complete ROIs between recurrent and non-recurrent subregions in NPC treated with radiotherapy. Following a high success rate in risk stratification using MR-based radiomic features, our results revealed that conventional MR-based radiomics might contain information with a similarly high impact on prognosis as angiogenesis information in accordance with DCE parameters. The above results suggest that MR-based radiomics feature maps can not only potentially visualize the heterogeneity and angiogenesis of the tumor from the ROI patches, but also allow tracing of the most revealing sub-regions in the analysis of a radiomics model.

Radiomics can be an effective method for monitoring phenotypic changes associated with prognosis in clinical settings. Our results revealed that radiomic features contained useful prognostic information in patients with NPC, and that the radiomics feature maps could visualize the heterogeneity and angiogenesis information of tumors, thereby improving the interpretation ability of radiomics. These results suggest that radiomics can decode the general phenotype associated with NPC prognosis. However, the conventional MR-based radiomics feature map showed similar but incomprehensive heterogeneity compared to the DCE parameter maps. A comparison between the MR-based radiomics feature map and the four pharmacokinetic parameters of DCE-MR images showed that not all the angiogenesis information on DCE-MR could be reflected by the radiomic features, which may explain why the integration of DCE-MR features and MR-based radiomics model prediction can significantly improve prognostic performance. Note that in the process of generating feature map, an essential observation is that the tumor ROI dimensions dictate the maximum window width during the feature map creation. Hence, an impending challenge lies in developing methods that assess the fidelity of model-used features, particularly at this maximum window width.

This study has several limitations. First, despite our efforts to include an exhaustive patient database, elevated censoring rates may have biased our findings. As a requirement for robustness, it is paramount to further validate our model against a dataset enriched with more extensive data. Second, the paucity of DCE-MR patients relative to the abundance of radiomic features necessitates meticulous feature selection to avoid overfitting. As the DCE-MR patient cohort expands in future work, there is the potential to incorporate more radiomic features. This would allow us to mine comprehensive prognostic prediction information, thereby enhancing the performance of our model. Third, the widespread use of DCE-MR came much later than that of conventional MR, resulting in a significant difference in follow-up between the MR and multi-modality cohorts. The drastically shorter follow-up of the DCE-MR cohort may harm the ability to build a convincing model to some extent. More DCE-MR-based radiomics studies with longer follow-up periods should be conducted. Fourth, although the VIF analysis was performed on the final selected set of features to mitigate the impact of multicollinearity on the final model, the regression models constructed during the feature selection process were still susceptible to multicollinearity. In this study, we followed common practices observed in previous research; however, it is important to optimize this aspect in future studies. Finally, given that certain studies indicate that NRIs in the context of inadequately fitted risk functions can at times be misleading [47], we urge readers to approach conclusions regarding the NRIs in our study with due circumspection.

## Conclusions

A combined radiomics model was identified by integrating DCE-MR and MR, which outperformed conventional MR-based radiomics models and can be used as an artificial intelligence tool for individualized prognostic assessment before treatment in patients with NPC.

### Supplementary Information

**Additional file 1.**

## Data Availability

The datasets used and analyzed in the current study are available from the corresponding author upon reasonable request.

## Abbreviations

MR: Magnetic resonance
DCE-MR: Dynamic contrast-enhanced magnetic resonance
NRI: Net reclassification index
LASSO: Least absolute shrinkage and selection operator
NPC: Nasopharyngeal carcinoma
IQR: Interquartile range
CET1-w: Contrast-enhanced T1-weighted
PFS: Progression-free survival
TNM: Tumor-node-metastasis
*v*_*e*_: Volume fraction of extravascular extracellular space
*v*_*p*_: Volume fraction of plasma space
*K*^*trans*^: Volume transfer constant
*k*_*ep*_: Reverse reflux rate constant
AIF: Arterial input function
ROI: Region of interest
PD-w: Proton density-weighted
CPH: Cox proportional hazards
VIF: Variance inflation factor
LAHGLE: Large-area high-gray-level emphasis
