# Supplementary material for: Comprehensive integrated analysis of MR and DCE-MR radiomics models for prognostic prediction in nasopharyngeal carcinoma
Source: Vis Comput Ind Biomed Art. 2023 Dec 1;6:23. doi: 10.1186/s42492-023-00149-0 (PMC10689317; doi:10.1186/s42492-023-00149-0)
Supplement: Supplementary file 1 — Additional file 1. [file 42492_2023_149_MOESM1_ESM.docx]

**Supplementary Methods**

# 1. Inclusion and exclusion criteria

All the patients were eligible according to the following inclusion criteria: (1) biopsy and histopathology confirmed NPC; (2) having Karnofsky performance status ≥70%; (3) receiving comprehensive treatment based on IMRT; (4) having pretreatment MR images. Patients were excluded according to the following exclusion criteria: (1) had other malignant tumors; (2) had radiotherapy or MRI contraindications (e.g., cardiac pacemaker implantation); (3) had previously received neck-based radiotherapy; (4) were pregnant or breastfeeding; (5) suffered from severe neurological diseases.

# 2. MRI scanning protocol

The protocol included a turbo spin echo (TSE) sequence was performed to obtain axial proton density-weighted (Pd-w) images (TR=4070 ms, TE=30 ms, FOV = 180×180 mm^2^ , matrix=384×384, and 4.0 mm slice thickness), and a fast low angle shot (FLASH)/vibe sequence for the acquisition of DCE including 50 dynamic acquisitions, 4.9 s per dynamic acquisition, with the following parameters: TR = 4.09 ms, TE = 1.47 ms, flip angle = 9°, phase = 75%, bandwidth = 400 Hz, thickness = 4 mm, slice gap = 0 mm, FOV = 180×180 mm^2^ , matrix = 192×144, TA = 245 s. Another FLAS/vibe sequence was performed to obtain T1-mapping before DCE-MR with 5 different flip angles, including 3°, 6°, 9°, 12°, and 15°. All other parameters were the same with the DCE. The contrast agent Gadodiamide (Omniscan, GE Medical Systems, Amersham, Ireland) was administered intravenously during the third dynamic acquisition using a power injector system (Spectris Solaris, MedRad, Indianola, PA, USA) at 0.1 mmoL/Kg body weight and 2 mL/sec, immediately followed by a 25-ml saline flush at a rate of 3.5 mL per second. A spin echo (SE) sequence for the acquisition of axial contrast-enhanced T1-weighted (CET1-w) images (repetition time (TR) = 625 ms, echo time (TE)=9.0 ms, field of view (FOV)=180×180 mm^2^, matrix=256×256, and 4.0 mm slice thickness).

# Radiomics feature engineering

**3.1 Image processing information**

a. Image resampling.

In this study, all the MR images and corresponding segmentation masks were interpolated with B-spline interpolation algorithm to have a uniform pixel spacing of 3.0×3.0×3.0 mm3, which helped ensure a common spatial resolution for the reproducibility of feature values.

b. Image intensity normalization and discretization.

Since the MR signal is usually relative, with large differences between scanners and vendors, we normalized the image on all gray values (not just those in the ROI), with a normalized scale of 100. Then, we performed gray value discretization by using a window width of 5 intensity values, and the lowest intensity in the first bin was 0 (after normalization).

c. Image filter.

A total of 8 filters were used to generate the derived images from the original images, including: Laplacian of Gaussian (LoG) filter, wavelet filter, square filter, square root filter, logarithm filter, exponential filter, gradient filter, and local binary pattern filter. Herein, the LoG filter was an edge enhancement filter with the width of the Gaussian kernel (σ) set to 1.0, 3.0, and 5.0, where σ could emphasize more fine (low σ values) or coarse (high σ values) textures. The wavelet filter yielded eight decompositions by applying either a High or a Low pass filter in each of the three dimensions. Moreover, the image filtering was performed before interpolation.

d. Image processing of pharmacokinetic maps and radiomics feature map

The pre-processing process of pharmacokinetic maps was essentially the same as that of MR images. Specifically, all the pharmacokinetic maps and corresponding segmentation masks were interpolated with B-spline interpolation algorithm to have a uniform pixel spacing of 3.0×3.0×3.0 mm3. Before discretizing the Pharmacokinetic maps, we also normalize them to a normalization scale of 100, and performed gray value discretization by using a window width of 5 intensity values. Eight filters (Laplacian of Gaussian, wavelet, square, square root, logarithm, exponential, gradient, and local binary pattern) were employed to derive images from original images. The LoG filter was an edge enhancement filter with a Gaussian kernel width (σ) of 1.0, 3.0, and 5.0, able to enhance textures of varying fineness. The wavelet filter produced eight decompositions using High or Low pass filtering in three dimensions.

In our radiomics feature map calculation, we implemented an identical pre-processing methodology as for MR imaging. However, during feature extraction, we exclusively derived finalized MR image radiomic features, specifically lbp_firstorder_Variance and wavelet-LHL_glszm_LAHGLE in the PD-w sequence, and wavelet-HHH_glszm_LAHGLE and squareroot_firstorder_RMAD in the CET1-w sequence. This selective feature extraction approach was designed to streamline computation of the feature map. In other words, prior to selecting the window images from the MR images, resampling, intensity normalization, and discretization based on the MR images have been performed.

**3.2** **The selected MR radiomics features in MR-based model**

Details of 4 MR radiomics features selected in this study are as follows:

“***wavelet-LHL_glszm_LargeAreaHighGrayLevelEmphasis(******LAHGLE)***”: Large area high gray level emphasis (LAHGLE) is calculated from gray level size zone matrix based on Pd-w image. “LHL” represents the wavelet decompositions by using two low-pass filters and one high-pass filter in three directions of the original image respectively. Similarly, “***wavelet-HHH_glszm_LAHGLE*** ” is calculated based on CET1-w image, and “HHH” represents the wavelet decompositions by using three high-pass filters in three directions. The calculation formula of LAHGLE is as follows.

$$LAHGLE= \frac{\sum_{i=1}^{N_{g}} \sum_{j=1}^{N_{s}} P(i,j)i^{2}j^{2}}{N_{z}}$$

*Where:*

$P(i,j)$ is the size zone matrix

$N_{g}$ is the number of discreet intensity values in the image

$N_{s}$ is the number of discreet zone sizes in the image

$$N_{z}= \sum_{i=1}^{N_{g}} \sum_{j=1}^{N_{s}} P(i,j)i^{2}j^{2}$$

"***lbp_firstorder_Variance***": First-order statistical feature based on the Pd-w images, " lbp_firstorder_Variance " is calculated from local binary pattern (LBP) derived images. Variance is the mean of the squared distances of each intensity value from the Mean value. This is a measure of the spread of the distribution about the mean. The calculation formula is as follows.

$$\mathrm{Variance}=\frac{1}{N_{p}}\sum_{i=1}^{N_{p}} {(X\left( i \right)-\bar{X})}^{2}$$

*Where:*

$N_{p}$ is the number of voxels within the ROI

X is a set of $N_{p}$ voxels included in the ROI

"***squareroot_firstorder_RobustMeanAbsoluteDeviation***": Robust mean absolute deviation (RMAD) is calculated from CET1-w image, and is the mean distance of all intensity values from the mean value calculated on the subset of image array with gray levels in between, or equal to the 10th and 90th percentile. The calculation formula is as follows.

$$\mathrm{RMAD}=\frac{1}{N_{10-90}}\sum_{i=1}^{N_{10-90}} \left| X_{10-90}\left( i \right)-\bar{X_{10-90}} \right)|$$

**3.3 The selected features in multi-parameter DCE-MR based model**

Details of 3 DCE-MR radiomics features selected in multi-parameter DCE-MR based model are as follows:

“***ve_wavelet.HLH_ngtdm_Busyness***”: Extracted from $V_{e}$, a measure of the change from a pixel to its neighbor. A high value for busyness indicates a ‘busy’ image, with rapid changes of intensity between pixels and its neighborhood. “HLH” represents the wavelet decompositions by using one high-pass filter, low-pass filter, and high-pass filter in three directions of the original image respectively. The calculation formula is as follows.

$$Busyness=\frac{\sum_{i=1}^{Ng} p(i)s(i)}{\sum_{i=1}^{N_{g}} \sum_{j=1}^{N_{g}\left| i p(i)-j p(j) \right|}}$$

*Where:*

$$p(i)\neq0, p(j)\neq0$$

$$p(i)=\sum_{j=1}^{N_{g}} p(i,j)$$

$P\left( i,j \right) be the co$-$occurence matrix$

***“******vp_wavelet.HHH_gldm_LargeDependenceHighGrayLevelEmphasis”***: Extracted from $V_{p}$, large dependence high gray level emphasis is calculated from gray level dependence matrix based on $V_{p}$ image of DCE-MR. “HHH” represents the wavelet decompositions by using three high-pass filter in three directions of the original image respectively. It measures the joint distribution of large dependence with higher gray-level values. The calculation formula is as follows.

$$LDHGLE= \frac{\sum_{i=1}^{N_{g}} \sum_{j=1}^{N_{d}} P(i,j)i^{2}j^{2}}{N_{z}}$$

*Where:*

$$P\left( i,j \right) be the dependence matrix$$

$$N_{z}=\sum_{i=1}^{N_{g}} \sum_{j=1}^{N_{d}} P(i,j)$$

$$N_{g} is the number of discrete intensity values in the image$$

$$N_{d} is the number of discrete dependency sizes in the image$$

***“******ktrans_lbp.2D_glszm_GrayLevelVariance”***: Extracted from *K^trans^*, measures the variance in grey level in the image. The calculation formula is as follows.

$$GrayLevelVariance=\sum_{i=1}^{N_{g}} \sum_{j=1}^{N_{d}} p(i,j)(i-\mu)^{2}$$

*Where:*

$$\mu=\sum_{i=1}^{N_{g}} \sum_{j=1}^{N_{d}} ip(i,j)$$

$P(i,j)$ is the size zone matrix

$N_{g}$ is the number of discreet intensity values in the image

$N_{d}$ is the number of discrete dependency sizes in the image

**3.4 The selected DCE-MR radiomics features in combined model**

Details of 4 DCE-MR radiomics features selected in combined model are as follows:

"***ktrans_wavelet.HLL_glcm_MCC***": Maximal correlation coefficient (MCC) is calculated from gray level co-occurrence matrix based on *K^trans^* image of DCE-MR. It’s a measure of complexity of the texture. “HLL” represents the wavelet decompositions by using one high-pass filter and two low-pass filters in three directions of the original image respectively. The calculation formula is as follows.

$$MCC=\sqrt{second largest eigenvalue of Q}$$

*Where:*

$$Q\left( i,j \right)=\sum_{k=0}^{N_{g}} \frac{p(i,k)p(j,k)}{p_{x}(i)p_{y}(k)}$$

$$p\left( i,j \right)=\frac{P(i,j)}{\sum P(i,j)}$$

$$p_{x}(i)=\sum_{j=1}^{N_{g}} p(i,j)$$

$P\left( i,j \right) be the co$-$occurence matrix$

"***ktrans_lbp.2D_glszm_GrayLevelNonUniformityNormalized***": Gray level non-uniformity normalized (GLNN) is calculated from gray level size zone matrix based on *K^trans^* image of DCE-MR. It measures the variability of gray-level intensity values in the image. The calculation formula is as follows.

$$GLNN=\frac{\sum_{i=1}^{N_{g}} {(\sum_{j=1}^{N_{s}} P(i,j))}^{2}}{N_{z}^{2}}$$

*Where:*

$P(i,j)$ is the size zone matrix

$N_{g}$ is the number of discreet intensity values in the image

$N_{s}$ is the number of discreet zone sizes in the image

$$N_{z}= \sum_{i=1}^{N_{g}} \sum_{j=1}^{N_{s}} P(i,j)i^{2}j^{2}$$

"***kep_wavelet.HHL_ngtdm_Complexity***": Complexity is calculated from neighboring gray tone difference matrix based on *K^ep^* image of DCE-MR. An image is considered complex when there are many primitive components in the image. The calculation formula is as follows.

$$Complexity=\frac{1}{N_{v,p}}\sum_{i=1}^{N_{g}} \sum_{j=1}^{N_{g}} |i-j|\frac{p_{i}s_{i}+p_{j}s_{j}}{p_{i}+p_{j}}$$

*Where:*

$N_{v,p}=\sum n_{i}$

$$s_{i}=\left\{ \begin{aligned} \sum_{n_{i}} |i-\bar{A}_{i}| for n_{i}\neq0 \\ 0 for n_{i}=0 \end{aligned} \right.$$

$$p_{i}=\frac{n_{i}}{N_{v}}$$

$$p_{i}\neq0, p_{j}\neq0$$

$$N_{g} be the number of discrete gray levels$$

$$n_{i} be the number of voexels in a set of segmented voxels$$

"***kep_wavelet.HHH_gldm_LargeDependenceHighGrayLevelEmphasis***": Large dependence high gray level emphasis is calculated from gray level dependence matrix based on *K^ep^* image of DCE-MR. “HHH” represents the wavelet decompositions by using three high-pass filter in three directions of the original image respectively. The formula can be can be referred to the “vp_wavelet. HHH_gldm_LargeDependenceHighGrayLevelEmphasis” in section 3.3.

More detailed information about radiomics feature engineering can be found in Pyradiomics project online documentation (<https://pyradiomics.readthedocs.io/>, version: 3.0.1).

# 3.5 Feature selection process of radiomics models

For the construction of the combined model, the features from the best MR and best DCE-MR based models were not directly used. On the one hand, feeding all the features in the MR and DCE-MR based models feature sets respectively to construct the combined model may lead to high correlation among features. On the other hand, since the MR-based model outperforms the DCE-MR-based model, our motivation for constructing the combined model is to test whether the DCE-MR-based features can further improve the MR-based model in prognostic prediction. Therefore, we use the output of the optimal MR based model and the DCE-MR based radiomic features as the initial feature set for feature selection. The detailed feature selection process is shown in the figure below.


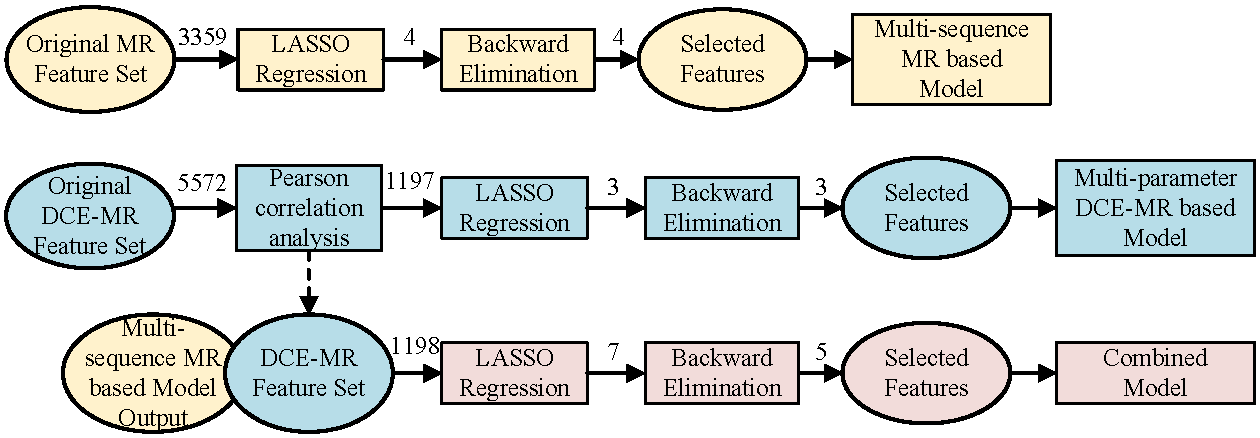


# 3.6 LASSO-CV based feature selection process


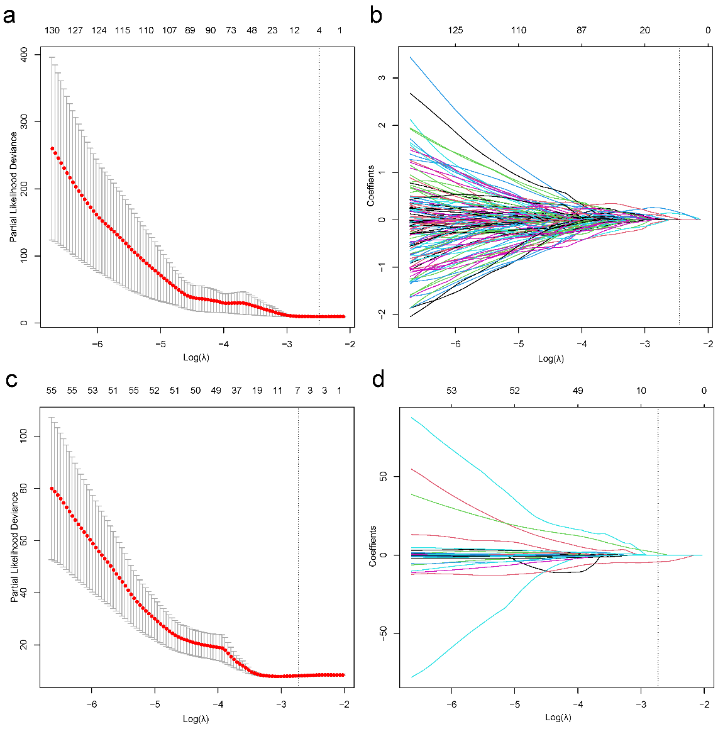


The LASSO-CV based feature selection process of the multi-sequence MR based model (a and b) and the combined model (c and d). Sub-figures (a) and (c) show the selection of tuning parameter (λ), herein the LASSO method uses 10-fold cross-validation via minimum error criteria. Sub-figures (b) and (d) show the LASSO coefficient profiles produced against the log (λ) sequence. Specifically, a λ sequence to be validated is initially obtained. Subsequently, 10-fold cross-validation is performed for each λ value on the training set (without utilizing the independent hold-out validation set). This process calculates the average and variance of the Partial-likelihood deviance for each candidate λ (as illustrated in the figure above). Finally, the optimal tuning parameter λ is determined based on the minimum average Partial-likelihood deviance.

# 3.7 Results of multicollinearity analysis

| Model | Range of VIF |
| --- | --- |
| PD-w | (1.005-1.006) |
| CET1-w | (1.037-1.147) |
| Multi-sequence MR | (1.014-1.305) |
| *v_e_* | (1.237-4.249) |
| *v_p_* | (1.098-1.099) |
| *K^trans^* | (1.067-1.138) |
| *k_ep_* | (1.012-1.031) |
| Multi-parameter DCE-MR | (1.014-1.375) |
| Combined Model | (1.129-1.457) |

# 4.Radiomics feature map

At the patch level, we re-extracted the 4 radiomics features selected from PD-w and CET1-w sequences in the MR based model, respectively. Then, the patch-based feature values were concatenated according to the patch coordinates to obtain the final radiomics feature map. Since the sliding window algorithm will slightly change the size of the feature map, we resized it to the size of the original image.

Due to the existence of a large number of patches in each image, a large amount of computing time will be consumed when we calculate radiomics features for each patch. On one hand, the multiprocessing pool in Python was used to accelerate the computation, which can reduce computation time by at least 2 times, depending on the number of CPU cores. On the other hand, we only performed slide-window algorithm on tumor areas in the original image according to ROI.
